# Editorial: Women in hypertension

**DOI:** 10.3389/fcvm.2023.1156589

**Published:** 2023-03-24

**Authors:** Maria Dorobantu, Daniela Sorriento

**Affiliations:** ^1^ Department of Cardiology, “Carol Davila” University of Medicine and Pharmacy, Bucharest, Romania; ^2^ The Romanian Academy, Bucharest, Romania; ^3^ Department of Advanced Biomedical Sciences, Federico II University, Naples, Italy; ^4^ CIRIAPA Interdepartmental Center for Research on Arterial Hypertension and Associated Conditions CIRIAPA, Federico II University, Naples, Italy

**Keywords:** hypertension, women, preeclampsia, anti-hypertensive treatment, risk-prediction model, gender discrimination

**Editorial on the Research Topic** Women in hypertension

## Introduction

Women's history in science has always been a history of prejudice and discrimination, without equality with the opposite sex. Despite the great progresses, such discrimination is still present in the scientific world, especially in the so-called hard sciences (Mathematics, Physics, Chemistry, Biology), maybe due to old gender prejudices and stereotypes. In the academic world, female researchers currently represent more than half of all researchers, but this percentage decreases drastically as we advance in the university hierarchy, predisposing to a clear gender inequality. In the leading academic positions, gender differences are even more marked, denoting an unfair approach towards female leadership and an overall aged attitude that does not reflect the current educational status. Although history does not give much credit to female researchers, many scientific discoveries belong to women. Over time, various female personalities have made scientific history and have proven to be a source of inspiration for future generations. A famous example is Marie Curie, one of the first scientists recognized worldwide for her studies on radiation and radioactive materials (Nobel Prizes for Physics in 1903 and Chemistry in 1911). The story of Rosalind Franklin is yet another emblematic one since she created the foundations of molecular biology by providing experimental evidence of the helix structure of DNA, even though the Nobel Prize was later awarded to her male colleagues. Francoise Barre-Sinoussi was awarded the Nobel Prize for Medicine in 2008 following the discovery of the human immunodeficiency virus (HIV), essential to turn AIDS from a death sentence to a manageable disease. Rita Levi-Montalcini received the Nobel Prize for Medicine in 1986 for the identification of the nerve fiber growth factor Ngf contributing to the study of several diseases, such as tumors and Alzheimer's disease.

Several projects aimed at gender equality are currently being developed, including this specific research topic, to promote female research, encourage women to be involved in scientific projects and, eventually, disseminate their results. Nevertheless, despite the progress that we have seen in recent years, gender equality is still far from being achieved. Sensitivity towards this problem has certainly grown and several initiatives are increasingly successful in promoting the much-needed cultural change.

## Contributions to the topic

The current Research Topic, entitled “Women in hypertension”, promotes the work of female scientists in the field of hypertension contributing to counteracting the gender imbalance currently present in the research field. To support this purpose, both the editors and the reviewers of this Editorial are women, and only submissions headed by women (as first or last author) were considered. In this context, the editors themselves are an example of female leadership in pre-clinical and clinical research fields: the SEPHAR study, led by Prof. Maria Dorobantu, had a major impact on the actual understanding of overall cardiovascular disease (1–3); pre-clinical research, led by Prof. Sorriento, increased the knowledge of endothelial function in diseases (4–9). To further emphasize the importance of this research topic, we have ultimately selected nine scientific studies, which significantly contributed to advances in the field of hypertension, the most common modifiable risk factor for cardiovascular and other diseases (10, 11). Risk prediction and an early diagnosis of hypertension are essential for the primary prevention and management of this condition and its cardiovascular complications. Therefore, effective, and easy-to-manage hypertension risk prediction models (machine learning models) have been generated to identify individuals at high risk of developing hypertension (12, 13). Practice guidelines are available for the management of hypertension, indicating the most effective drugs, therapeutic associations, and lifestyle modifications (14–15) to prevent cardiovascular events and reduce mortality, but many patients with hypertension remain uncontrolled. This could be partly due to diagnostic and treatment initiation inertia (16) and the type of intervention and clinical approach (17). Novel therapeutic targets have been identified using pre-clinical models of hypertension but most failed in clinical trials or generated contrasting results, possibly due to defects in patient enrollment and comorbidities, as it occurs with Vitamin D supplementation (18–21). The gender difference in blood pressure levels appears during adolescence and in the elderly (22). Premenopausal women have a lower risk and incidence of hypertension compared with men of the same age, but this advantage for women gradually disappears after menopause. In this context, clinical and experimental findings emphasize the role of sex hormones, the autonomic nervous system, the renin-angiotensin-aldosterone system, and arterial stiffness in the development of chronically elevated blood pressure in women (24). A particular condition that requires special attention from physicians is preeclampsia, the leading cause of maternal and neonatal death, for which an early diagnosis and a timely initiated and well-conducted antihypertensive therapy are essential (25–27). With the sole exception of pregnancy, the current good clinical practice guidelines do not make any differences between men and women regarding the general therapeutic approach (28), even if a gender-related response to therapy has been suggested (29–31).

## Conclusions

The contributions of this Research Topic from female researchers demonstrate the remarkable contribution of females in the research field, contributing to advances in knowledge of hypertension. In the future, female participation in the scientific field and their access to leading positions in academia and science should be further encouraged and supported. In a society where we are aiming at overall transparency and fairness, where our only purpose should be notable progress in medicine and science regardless of the subspecialty, discrimination based on the researcher's gender has no place. The world has no gender but only brilliant minds at the service of science!
